# Prognostics and Health Management of Industrial Assets: Current Progress and Road Ahead

**DOI:** 10.3389/frai.2020.578613

**Published:** 2020-11-09

**Authors:** Luca Biggio, Iason Kastanis

**Affiliations:** ^1^ Data Analytics Lab, Institute of Machine Learning, Department of Computer Science, ETHZ: Eidgenössische Technische Hochschule Zürich, Zurich, Switzerland; ^2^ Robotics and Automation, CSEM SA: Swiss Center for Electronics and Microtechnology S.A., Alpnach, Switzerland

**Keywords:** prognostic and health management, predictive maintenance, industry 4.0, artificial intelligence, machine learning, deep leaning

## Abstract

Prognostic and Health Management (PHM) systems are some of the main protagonists of the Industry 4.0 revolution. Efficiently detecting whether an industrial component has deviated from its normal operating condition or predicting when a fault will occur are the main challenges these systems aim at addressing. Efficient PHM methods promise to decrease the probability of extreme failure events, thus improving the safety level of industrial machines. Furthermore, they could potentially drastically reduce the often conspicuous costs associated with scheduled maintenance operations. The increasing availability of data and the stunning progress of Machine Learning (ML) and Deep Learning (DL) techniques over the last decade represent two strong motivating factors for the development of data-driven PHM systems. On the other hand, the black-box nature of DL models significantly hinders their level of interpretability, *de facto* limiting their application to real-world scenarios. In this work, we explore the intersection of Artificial Intelligence (AI) methods and PHM applications. We present a thorough review of existing works both in the contexts of fault diagnosis and fault prognosis, highlighting the benefits and the drawbacks introduced by the adoption of AI techniques. Our goal is to highlight potentially fruitful research directions along with characterizing the main challenges that need to be addressed in order to realize the promises of AI-based PHM systems.

## 1 Introduction

Supporting the constant growth of modern industrial markets makes the optimization of operational efficiency and the minimization of superfluous costs essential. A substantial part of these costs often derives from the maintenance of industrial assets.

Recent studies^1^ show that, for the average factory, inefficient maintenance policies are responsible for costs ranging from 5 to 20% of the plant’s entire productive capacity. Furthermore, according to the International Society of Automation (ISA)^2^, the overall burden of unplanned downtime on industrial manufacturers across all industry segments is estimated to touch the impressive figure of $647 billion per year.

If, on one hand, the above considerations highlight the fundamental impact of maintenance operations on manufacturers’ balances, on the other hand a large number of companies are still not satisfied with their maintenance strategies. According to a recent trend study gathering interviews with more than 230 senior European business^3^, roughly 93% of them deem their maintenance policy inefficient.

As discussed later, the current most popular approaches to maintenance are divided into two categories, namely reactive maintenance and scheduled maintenance. Roughly speaking, the first implements maintenance operations immediately after a system failure occurs, whereas the second is based on scheduling maintenance operations at regular time intervals. These strategies naturally introduce significant extra costs due to machine downtime, component replacement or unnecessary maintenance interventions.

On the other hand, Predictive Maintenance (PM) represents a completely different paradigm that holds the promise of overcoming the inefficiencies of the aforementioned methods. PM is one of the hallmarks of the so-called Industry 4.0 revolution, i.e., the process of modernization of the industrial world induced by the advent of the digitalization era. The goal of PM systems is to implement a smarter and more dynamical approach to maintenance leveraging recent advances in sensor engineering and data analysis. The health state of a machine is now constantly monitored by a network of sensors and future maintenance operations are based on the analysis of the resulting data. An increasing number of organizations, motivated by their need for reducing costs and by the potential of PM, are starting to invest significant amounts of resources on the modernization of their current maintenance strategies^1^.

One natural question arising now is to what extent PM solutions can actually improve a company’s efficiency in terms of reduction of downtime, cost savings and safety. A recent PWC study^4^ investigates the actual potential of PM beyond the hype generated around it in the last few years. The results are quite impressive: 95% of the interviewed organizations claim that the adoption of PM strategies contributed to the improvement of several key performance indicators. Roughly 60% of the involved companies report average improvements of more than 9% of machines uptime, and further enhancements in terms of cost savings, health risks, assets lifetime.

As mentioned above, as a key player in the fourth industrial revolution, PM exploits some of the most recent advances introduced in the last few years in computer science and information engineering. Among them, ML is arguably one of the technologies that is experiencing the most impressive growth in terms of investments and interest of the private sector. This increasing attention in AI technologies is mainly due to the tremendous contributions they have brought in fields such as Computer Vision (CV), Natural Language Processing (NLP) and Speech Recognition in the last decade.

PM approaches are heavily based on ML techniques. The increasing availability of relatively cheap sensors has made much easier to collect large amounts of data, which are in turn the main ingredients ML systems necessitate.

However, AI-based technologies should not be considered as a “silver bullet” capable of immediately addressing all the issues affecting current maintenance strategies. ML and DL, in particular, are constantly evolving fields and, despite their significant achievements, a number of drawbacks still limit their wide application to real-world scenarios. It is, therefore, necessary to be cautious and try to understand the limitations of current AI approaches in the context of PM and drive further research toward the resolution or the alleviation of these shortcomings.

The goal of this manuscript is to provide an updated critical review of the main AI techniques currently used in the context of PM. Specifically, we focus on highlighting the benefits introduced by modern DL techniques along with the challenges that these systems are not yet able to solve. Furthermore, we present a number of relatively unexplored solutions to these open problems based on some of the most recent advances proposed in the AI community in the last few years.

This manuscript is structured as follows: Section 2 briefly describes classic maintenance strategies and introduces the core ideas from Prognostic and Health Management (PHM). Section 3 discusses the benefits of data-driven approaches and presents some of the most popular AI-based methods used in PHM. Section 4 summarizes the main open challenges in PHM and presents some of their possible solutions. Finally, Section 5 concludes the paper.

## 2 Elements of Prognostic and Health Management

Prognostic and Health Management (PHM) is an engineering field whose goal is to provide users with a thorough analysis of the health condition of a machine and its components (Lee et al., 2014). To this extent, PHM employs tools from data science, statistics and physics in order to detect an eventual fault (anomaly detection) in the system, classify it according to its specific type (diagnostic) and forecast how long the machine will be able to work in presence of this fault (prognostic) (Kadry, 2012).

First, we present the most popular maintenance approaches, highlighting the advantages and disadvantages of these different methods in terms of costs and overall machine downtime. Then, we describe the entire PHM process by describing the role of its main sub-components in the context of the previously introduced maintenance approaches.

### 2.1 Different Approaches to Maintenance

The choice of an efficient maintenance strategy is crucial for reducing costs and minimizing the overall machine’s downtime. The adoption of a particular maintenance strategy primarily depends on the needs and the characteristics of the company’s production line. Indeed, each maintenance policy introduces some benefits and disadvantages directly impacting costs in different modalities. In this review, we identify four distinct approaches to maintenance, namely: Reactive Maintenance (RM), Scheduled Maintenance (SM), Condition-Based Maintenance (CBM), and Predictive Maintenance (PM) (Fink, 2020).

#### 2.1.1 Reactive Maintenance

RM consists of repairing or substituting a machine component only once it fails and it can no longer operate. The immediate advantage of this approach is that the amount of maintenance manpower and expenses related to keeping machines running are minimized (Swanson, 2001). Furthermore, since machines are active until they break, their utilization time is maximized. On the other hand, this approach is risky from many perspectives. First and foremost, it is potentially dangerous from the point of view of safety. Waiting for a machine to reach its maximum stress level can result in catastrophic failures. Moreover, this type of failures usually introduce larger costs and need a significant amount of time to be repaired. Therefore, by adopting this maintenance strategy, one might expect conspicuous costs arising both from reparations of severe failures and from relatively large unplanned machines downtimes.

#### 2.1.2 Scheduled Maintenance

**FIGURE 1:**
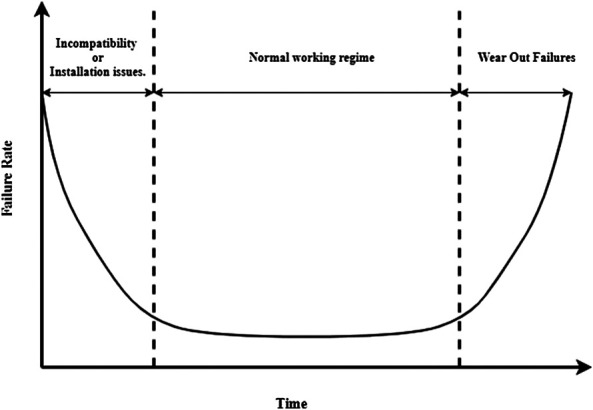
The bathub curve shows that the most likely times for a machine to break are right after the installation and after its normal operating time.

SM is based on maintenance interventions carried out at regular time intervals. The goal is to minimize the probability of failures and thus avoid costly unplanned downtimes by performing maintenance activities even when the machine is still operating under normal conditions. SM strongly relies on a meaningful schedule that has to be tailored to the specific properties of the equipment. In particular, experts have to provide a detailed evaluation of the failure behavior of the machines and of their components in order to maximize the level of accuracy on the prediction of the next failure time. This analysis typically results in the so-called “bathub” curves (Mobley, 2002), as shown in Figure 1.

The bathtub curve illustrated in Figure 1 shows that a machine component presents a high risk of failure right after it is installed (because of installation errors or incompatibility issues with other components) and after its normal operation interval (because of natural degradation and wear out.). Between these two phases, the machine is supposed to work properly and its failure probability is low and constant.

The main advantage of SM is that it significantly reduce unplanned downtime. Furthermore, the reparation costs are generally less dramatic than those encountered in RM, since, now, machines are not allowed to operate until their breaking point. On the other hand, a SM approach presents the concrete risk of carrying out several relatively expensive maintenance interventions even when the equipment is still working properly. Sticking to a fixed degradation model of a certain machine might lead maintenance operators to miss anomalies caused by external factors or internal malfunctions that make the machine’s degradation pattern deviate from its predicted trend.

#### 2.1.3 Condition Based and Predictive Maintenance

CBM and PM differ from the types of maintenance strategies previously described in that they employ data-driven techniques to assist technicians to efficiently set times for maintenance activities. The goal of these methods is to provide a good compromise between maintenance frequency and its relative costs (Ran et al., 2019).

The difference between CBM and PM lies entirely in their different responses when a defective system condition is detected. In this case, a CBM approach would intervene on the system immediately after the detection time. This method could lead to the replacement or repair of a component of the equipment even if it could have continued its normal routine for a longer time without affecting other parts of the machine. Furthermore, intervening immediately after the fault has been detected might result in stopping the machines’ working cycle at an inconvenient stage from the point of view of production efficiency.

To the contrary, PM tries to predict the useful lifetime of a component at a certain time step in order to indicate the point in the future where maintenance has to be performed. This last approach inevitably results in lower maintenance costs compared to CBM, since each component can be fully exploited without sacrificing safety and efficiency (Fink, 2020).

**FIGURE 2:**
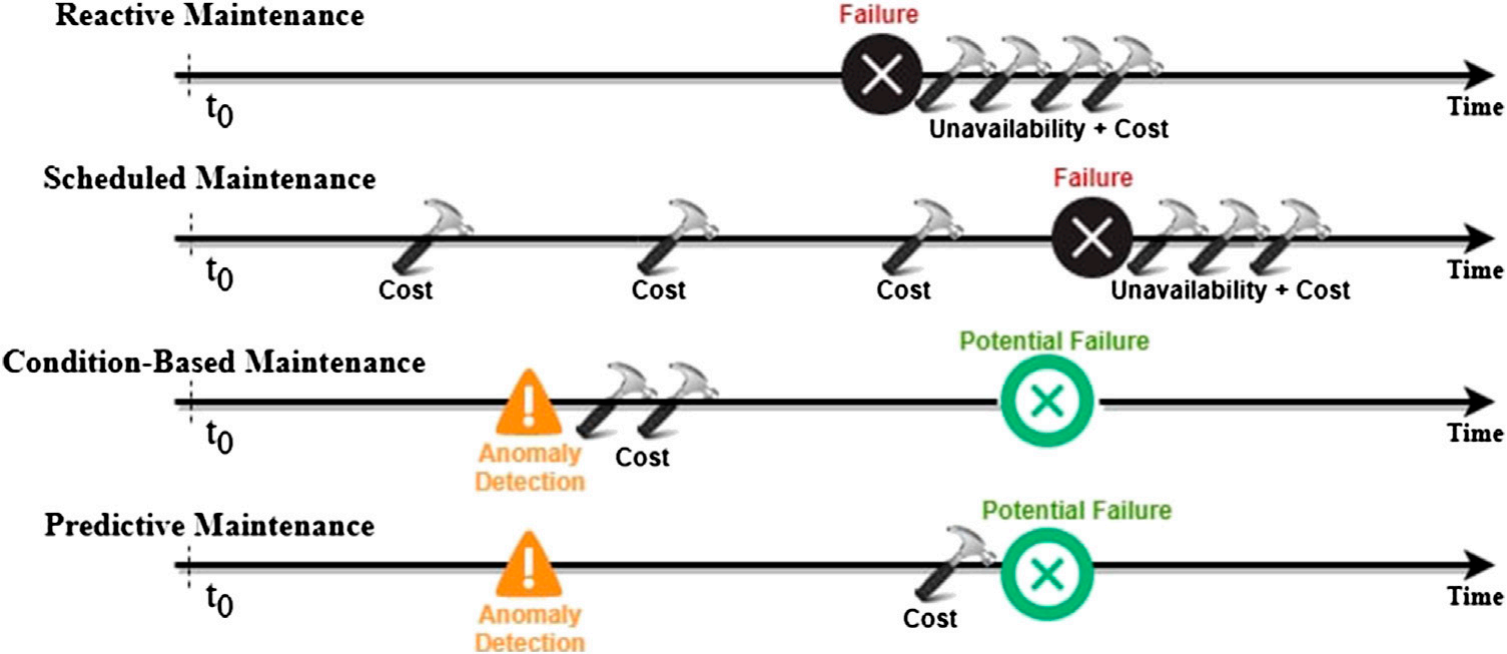
Scheme of the behavior of the different maintenance approaches described above. Figure adapted from Fink (2020).

Figure 2 summarizes the maintenance strategies presented above by illustrating the costs resulting from their different approaches.

### 2.2 Prognostic and Health Management Process

**FIGURE 3:**
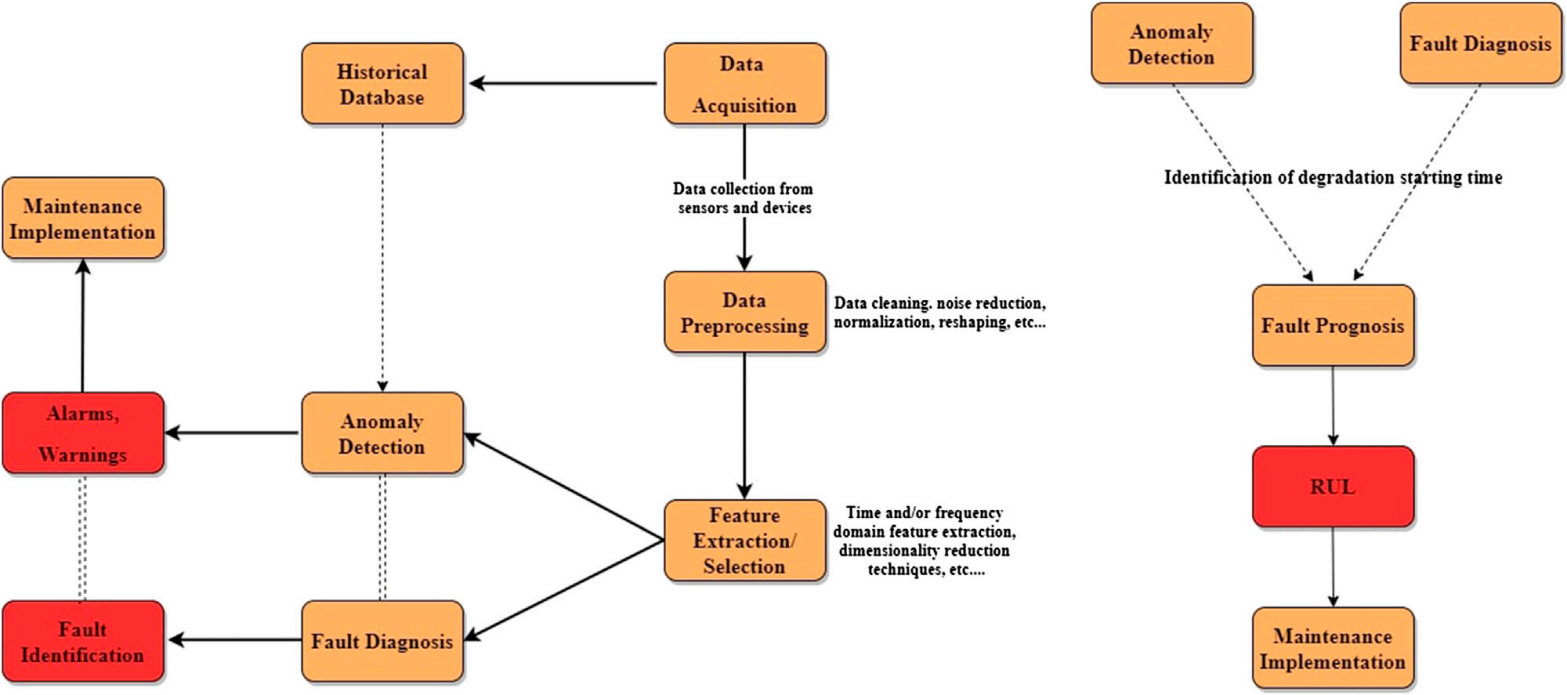
Main steps of the typical PHM process. This can be divided into CBM **(left)** and PM **(right)**. RUL estimation is enhanced by information extracted at the CBM level, such as the time step where degradation starts to show its effects. Figure adapted from Khan and Yairi (2018).

As mentioned before, PHM makes use of information extracted from data to assess the health state of an industrial component and driving maintenance operations accordingly. Figure 3 illustrates the main components constituting the typical PHM pipeline, from data acquisition to decision making.

The very first step of the PHM process consists of selecting a suitable set of sensors and devices, setting them up in the most appropriate location and deciding on an optimal sampling frequency for data collection. The communication system between sensors and databases must be implemented in order to allow for both real-time machine health monitoring and offline data handling. To this extent, a widely adopted solution by industries is the Open Platform Communication Unified Architecture (OPC UA), a popular communication protocol that allows information to be shared across sensors, industrial assets and the Cloud in a highly secure way (Bruckner et al., 2019).

Once the sensor array is in place, data can be acquired. These data are typically in forms that are not compatible with the input shape requested by AI algorithms. Therefore, a data pre-processing step must be implemented in order to clean the data, mitigate the effects induced by noise or simply reshape them so that their new format can be interpreted by data analysis techniques.

The resulting data are cleaner than the original ones but can still contain a substantial amount of redundant information. This motivates the application of feature extraction techniques to reduce the dimensionality of the data and retain only the most meaningful pieces of information. As we see in the next section, most modern AI techniques are designed to automatically extract informative features without any need for expert knowledge and manual feature engineering.

#### 2.2.1 Condition-Based Maintenance

CBM consists of two main elements: anomaly detection and diagnosis [see Figure 3 (left)]. Both these processes immediately follow the data extraction and data pre-processing pipelines described above and aim at supporting the decision making step with meaningful information about the state of the system. The information extracted by the anomaly detection and diagnosis modules can subsequently be exploited at the PM level in order to provide an even richer description of the machine’s health state [see Figure 3 (right)].

##### 2.2.1.1 Anomaly Detection

Anomaly detection is responsible for automatically establishing whether the input data present any discrepancy compared to some internal model of the normal machine’s behavior (Khan and Yairi, 2018). This internal representation can be learned by extracting and storing representative features from data gathered from healthy machines. It is important to note that, in general, healthy data, i.e., data gathered from machines working under normal working conditions, are much more abundant than faulty data. This is because, typically, a machine can incur in several different types of faults, each of which is, luckily, relatively rare. As a conclusive remark, we highlight that the detection of an anomaly does not necessarily imply that it corresponds to a fault. It might be, for instance, that it represents a new healthy feature that does not have any representatives into the historical data or has not been modeled by the anomaly detection algorithm’s internal model.

##### 2.2.1.2 Fault Diagnosis

Fault diagnosis moves one step forward with respect to anomaly detection since, besides detecting that an outlier is present, it also identifies the cause at the basis of that anomaly (Hess, 2002). Fault diagnosis models are based on historical data representing different faulty conditions. These data are used to characterize each type of fault and allow the models to classify new previously unseen data within a predefined set of fault cases.

#### 2.2.2 Predictive Maintenance

The main difference between CBM and PM is that PM algorithms deal with the problem of predicting the Remaining Useful Life (RUL) of an industrial component before a complete failure occurs and the machine is no longer able to operate (Medjaher et al., 2012; Fink, 2020). Therefore, the key enablers of PM strategies are algorithms capable of efficiently forecasting the future state of a machine, i.e., provide prognostic information about its RUL.

##### 2.2.2.1 Fault Prognosis

As mentioned before, fault prognosis is about providing an as accurate as possible prediction of the RUL of a certain machine component. The RUL estimation process starts from the identification of a time-step where a fault begins to show its effects. The final goal is to infer how long the machine can continue operating even in the presence of a degradation trend due to the previously detected fault.

Contrarily to diagnosis, time plays a crucial role in prognosis, since the objective is now to provide an estimate of the future time step when a certain event will occur (Lee et al., 2014). It is important to note that RUL predictions are strongly affected by various sources of noise. These can arise from noisy sensor readings, the inherent stochasticity of the RUL forecasting problem and the choice of an imperfect model for the machine degradation process.

## 3 Artificial Intelligence-Based Prognostic and Health Management

The attempt of devising artificial agents with the ability to emulate or even improve some aspects characterizing human intelligence is what makes AI an extremely exciting field of research both from a fundamental and a practical points of view. ML, as a branch of AI, studies the problem of designing machines capable of learning through experience and by extracting information from data (Mitchell, 1997). “Learning from experience” represents a distinctive human feature that enables us to actively interact with the world we live in. It allows us to build a progressively more accurate internal model of the surrounding environment by processing and interpreting the external signals our body is able to perceive.

Similarly to humans, intelligent systems can process the information perceived by an array of sensors about a given industrial component and provide a model of its operating condition and its health status. The increasing availability of data and the high level of computational power reached by modern hardware components make the application of AI techniques even more appealing.

ML has witnessed an increasing interest in the last few decades. A turning point has been set by the introduction of the first state-of-the-art DL technique almost 10 years ago by Krizhevsky et al. (2012) in the context of Image Recognition (IR). This event has triggered a new era in the field of data analysis characterized by a plethora of new applications of DL to a series of disparate engineering fields, ranging from NLP to CV.

The goal of this section is to give the reader an insight into the intersection of ML and PHM and the progress made by the scientific community hitherto. First, we present the main steps involved in the application of “traditional” ML techniques to PHM and we discuss how these can be utilized in the contexts of diagnosis and prognosis. Then, we present a number of popular DL techniques and we review some of their most interesting applications in this context.

### 3.1 “Classical” Machine Learning Methods

**FIGURE 4:**
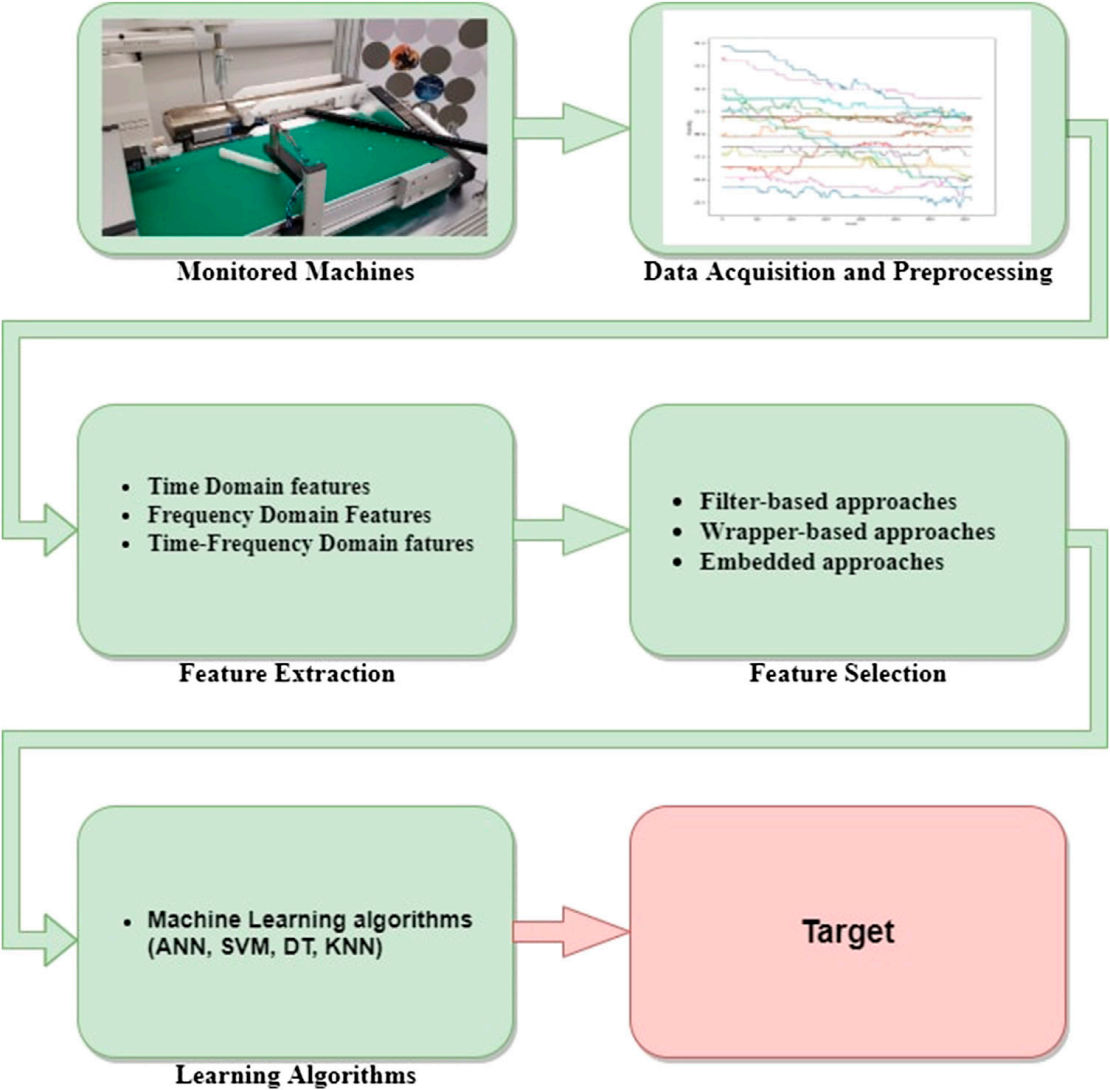
Main steps characterizing the approaches based on traditional ML algorithms. Adapted from Zhao et al. (2016).

Before the explosion of DL almost one decade ago, the typical process followed by the majority of data-driven approaches to PHM is illustrated in Figure 4. The raw measurements provided by a battery of sensors can not be straightforwardly linked with the health state of the machine or its RUL. Indeed, they are often affected by a significant amount of noise that can be introduced by either external factors, such as a sudden temperature increase, or imperfect signal transmissions. Furthermore, often these data are represented by complex time-series or images, that are typically characterized by a highly redundant information content that tends to hide the relatively limited discriminative features of interest. For the above reasons, once data are acquired, a set of candidate features have to be extracted and then, only the most informative among them have to be properly selected. Once these steps are completed, the final set of extracted features can be used to train a ML algorithm to perform the desired diagnosis or prognosis task.

In the following, we briefly go through all the aforementioned steps, discussing some of the main techniques involved in each of them.

#### 3.1.1 Feature Extraction and Feature Selection

##### 3.1.1.1 Feature Extraction

According to Yu (2019), feature extraction can be defined as the task of transforming raw data into more informative features that serve the need of follow-up predictive models and that help in improving performances on unseen data.

A general recipe for the feature extraction task does not exist and a set of key context-dependent factors must be taken into account. Some of these are, for example, the specific type of task to be performed, the characteristics of the data, the application domain and the algorithmic and efficiency requirement (Guyon et al., 2006). For instance, traditional choices of features in the context of IR are those obtained by the SIFT (Lowe, 2004) and SURF (Bay et al., 2008) algorithms, whereas mel-cepstral coefficients (Davis and Mermelstein, 1980; Kopparapu and Laxminarayana, 2010) are typically chosen in speech recognition applications.

In the context of PHM, data recorded for the purpose of equipment maintenance come often in the form of time-series. Therefore, an opportune set of features must be chosen according to the properties of the signals under consideration, e.g., its physical nature (temperature, pressure, voltage, acceleration,…), its dynamics (cyclic, periodic, stationary, stochastic), its sampling frequency and its sample value discretization (continuous, discrete)^5^. Typical examples of features extracted from raw time-series data can be divided into three categories (Lei et al., 2020): time domain, frequency domain and time-frequency domain. The first includes statistical indicators such as mean, standard deviation, root mean square, skewness, kurtosis, crest factor, signal-to-noise ratio. Other standard time-domain feature extraction methods are traditional signal processing techniques such as auto and cross-correlation, convolution, fractal analysis (Yang et al., 2007) and correlation dimension (Logan and Mathew, 1996). Finally, model-based approaches such as autoregressive (AR, ARMA) or probability distribution models where features consist of the model parameters (Poyhonen et al., 2004) are also commonly used.

Features extracted from the frequency domain are typically obtained through spectral analysis of the signal of interest. Fast-Fourier-Transform is applied to raw data to extract the power spectrum and retrieve information about the characteristic frequencies of the signal. Finally, time-frequency domain feature extraction techniques include short-time Fourier transform, wavelet transform and empirical mode decomposition, among others. The goal of these methods is to capture how the frequency components of the signal vary as functions of time and are particularly useful for non-stationary time-series analysis.

##### 3.1.1.2 Feature Selection

The goal of feature extraction is to obtain a first set of candidate features that are as informative as possible for the problem under consideration. Feature selection aims at reducing the dimension of the feature space by individuating a subset of features that are maximally relevant for a certain objective. According to the pioneering work of Guyon et al. (2006), feature selection methods can be divided into three categories: filters, wrappers and embedded methods. The first class of approaches consists of finding a subset of features that is optimal according to a specified objective measuring the information content of the proposed candidates. This objective is independent of the particular ML algorithm used to perform the PHM task and therefore the resulting features will be typically more general and potentially usable by different ML algorithms. Several feature selection techniques are based on the calculation of information-theoretic quantities such as the Pearson coefficient or the information gain. For instance, the Minimum-Redundancy-Maximum-Relevance (mRMR) technique is based on the idea that the optimal subset of features should be highly correlated with the target variable (which might be, for example, the classification label indicating a specific fault type) and mutually far away from each other.

Wrapper-based methods differs from their filter-based counterpart in the criteria they use for assessing the “goodness” of a specific set of features. Specifically, they directly employ the ML algorithm to get feedback, usually in form of accuracy or loss function, about the selected candidates. Wrappers are usually able to achieve better performances than filters since they are optimized with respect to a specific ML algorithm which is in turn tailored for a specific task. On the other hand, wrappers are biased toward the ML algorithm they are based on and therefore the resulting feature subset will not be generally adequate for alternative ML techniques.

The final class of feature selection methods is represented by the so-called embedded approaches. These techniques integrate the feature selection process directly into the ML algorithm in an end-to-end fashion. A popular example of embedded approach is the LASSO (Least Absolute Shrinkage and Selection Operator) (Tibshirani, 1996) which is a method for linear-regression that solves the following optimization problem:

$$\underset{w,b}{\text{min}}\frac{1}{n}\overset{n}{\underset{i=1}{\sum }}{\left(y_{i}-w^{T}x_{i}-b\right)}^{2}+\lambda w_{1}$$ (1)

with

$${\left|\left|w\right|\right|}_{1}=\overset{d}{\underset{j=1}{\sum }}|w^{\left(j\right)}|$$ (2)

The ${ℒ}^{1}$ norm forces the learnt solution $\hat{w}$ to be sparse and therefore, only the least redundant features are selected. Other methods used for end-to-end feature selection are, for instance, the Akaike Information Criterion (AIC) (Sakamoto et al., 1986) and the Bayesian Information Criterion (BIC) (Neath and Cavanaugh, 2012) which are both based on finding features that are generalizable and not problem-specific.

As a conclusive remark, it is worth mentioning that, similarly to feature selection approaches, also dimensionality reduction methods aim at reducing the level of redundancy and maximizing the amount of informativeness present among the feature candidates. Techniques such as Principal Component Analysis (PCA) (Jolliffe, 1986) are used to project data onto a lower-dimensional linear subspace perpendicular to the feature removed. Other popular dimensionality reduction techniques are Linear Discriminants Analysis (LDA) (McLachlan, 2004), Exploratory Projection Pursuit (EPP) (Friedman, 1987), Independent Component Analysis (ICA) (Hyvärinen and Oja, 2000) and T-distributed Stochastic Neighbor Embedding (t-SNE) (Maaten and Hinton, 2008), among others.

#### 3.1.2 Traditional Machine Learning Algorithms

As shown in Figure 4, once features are extracted and properly selected, they can be used as input for a ML algorithm responsible for performing the diagnosis or prognosis task we are interested in. In this section, we focus on “traditional” ML algorithms, i.e., popular AI methods widely employed before the advent of DL. These techniques can be divided into four main sub-categories, namely: (shallow) Artificial Neural Networks (ANNs), Support Vector Machines (SVMs), Decision Trees (DTs), and K-Nearest Neighbor (KNN).

##### 3.1.2.1 Diagnosis

All the aforementioned classes of algorithms have been applied to fault diagnosis in several different contexts. In the following, we first briefly discuss the basic principles of these methods and then we list some of their most interesting applications.

###### 3.1.2.1.1 Artificial Neural Networks

ANNs are popular ML algorithms whose design draws inspiration from the biological mechanism at the basis of neural connections in the human brain. They consist of elementary processing units, called neurons, connected to each other by means of dynamic weights of variable magnitudes, whose role is meant to emulate the behavior of synaptic connections in animals’ brains. Different types ANNs topologies can be constructed by differently organizing the neurons and their relative connections. The choice of the specific ANN architecture crucially depends on the nature of the task to be performed, the data structure under consideration and the availability of computational resources.

Over the last two decades, ANNs have been used to detect and classify faults incurring in several diverse types of machines. For instance, they have been applied to fault diagnosis of rolling element bearings (Samanta and Al-Balushi, 2003), induction motors (Ayhan et al., 2006), gears (Samanta, 2004; Abu-Mahfouz, 2005), engines (Lu et al., 2001), turbine blades (Kuo, 1995; Ngui et al., 2017), electrical (Moosavi et al., 2016) and photovoltaic (Chine et al., 2016) devices, among others.

The choice of output layer directly reflects the kind of task we are interested in. For instance, for fault detection tasks, two neurons can be used to output the probability that the input corresponds to a healthy instance or a faulty one. On the other hand, if we are interested in fault diagnosis, the number of output neurons is equal to the number of faults affecting the machine under consideration. A typical example of ANNs application to fault detection is provided by Samanta and Al-Balushi (2003). In this work, five time-domain features (RMS, skewness, variance, kurtosis, and normalized sixth central moment) are extracted from raw vibration signals. These features are then used as inputs to a shallow ANN consisting of two hidden layers with 16 and 10 neurons respectively and one output layer with two neurons (indicating if the input corresponds to normal or failed bearing).

###### 3.1.2.1.2 Support Vector Machines

Given a dataset ${\left\{x_{i},y_{i}\right\}}_{i=1}^{N}$, where $x_{i}\in {\mathbb{R}}^{d}$ and y=±1, SVMs aim at separating the two classes of data by finding the optimal hyperplane with the maximum margin between them. The margin is the distance between the nearest training data points of any class. In most real-world problem, data are not linearly separable. In these cases, the so-called kernel trick (Hofmann et al., 2008) can be used to tackle nonlinear classification tasks by implicitly mapping the data into a high-dimensional feature space.

Standard SVMs, along with a number of improved variants, have been extensively applied to fault diagnosis. For example, they have been used for assessing the health state of rolling element bearings (Yang et al., 2005; Abbasion et al., 2007; Gryllias and Antoniadis, 2012; Fernández-Francos et al., 2013; Islam et al., 2017; Islam and Kim, 2019b), induction motors (Widodo and Yang, 2007), gearboxes (Liu et al., 2013), engines (Li et al., 2012), wind turbines (Santos et al., 2015) and air conditioning systems (Sun et al., 2016a).

In order to perform fault diagnosis tasks, SVMs are typically employed alongside One-Against-One (OAO) (Yang et al., 2005; Islam et al., 2017) or One-Against-All (OAA) (Abbasion et al., 2007; Gryllias and Antoniadis, 2012) strategies. Furthermore, SVMs can also be applied to anomaly detection. For example, Liu et al. (2013) train a one-class SVM only on healthy data to detect anomalies in bearings vibrational data.

Generally, SVMs are particularly well suited for problems characterized by high-dimensional features. On the other hand, the computation of the N×N kernel matrix can be highly expensive when the number of data instances is relatively large.

###### 3.1.2.1.3 Decision Trees

Decision trees (DTs) represent a class of non-parametric supervised ML algorithms commonly used for regression and classification. DTs are trained to infer a mapping between data features and the corresponding output values by learning a set of relatively simple and interpretable decision rules. As the name suggests, these classification rules correspond to paths linking the root node to the leaf nodes. Indeed. each internal node can be seen as a condition on a particular attribute. The different outcomes of this test are represented by the branches generated from that node. The C4.5 algorithm (Quinlan, 2014) is one of the most popular approaches to learn a DT.

DTs have been widely employed in the context of fault diagnosis over the last two decades. For example, they have been applied to process data gathered from rolling element bearing systems (Sugumaran and Ramachandran, 2007; Sugumaran, 2012), gearboxes (Saravanan and Ramachandran, 2009; Praveenkumar et al., 2018), wind turbines (Abdallah et al., 2018), centrifugal pumps (Sakthivel et al., 2010), and photovoltaic systems (Benkercha and Moulahoum, 2018).

Multiple DTs can be employed jointly to form a random forest (RF), an ensemble learning algorithm capable of overcoming some shortcomings of single decision trees, such as limited generalization and overfitting. RFs have been successfully applied to fault diagnosis of induction motors (Yang et al., 2008), rolling bearings (Wang et al., 2017), and aircraft engines (Yan, 2006) among others.

The main advantages provided by DTs stand in their high level of interpretability, resulting from the easily decipherable decision rules they implement. Moreover, they often achieve reasonably high accuracies in most of the classification problems they are applied to. On the other hand, these methods are often prone to overfitting and therefore tend to provide poor generalization performances.

###### 3.1.2.1.4 K-Nearest Neighbor

KNN is non-parametric algorithm widely used for classification tasks. Given a set of input-output pairs ${\left\{x_{i},y_{i}\right\}}_{i=1}^{N}$ and a test datum $\hat{x}$, the KNN algorithm searches the *k* closest training inputs to $\hat{x}$ in the feature space and label the test datum with the label having more representatives among the *k* selected training data. Closeness can be measured by an arbitrary similarity measure, such as the Euclidean distance. Due to its simplicity and its high level in interpretability, KNN-based approaches have found many applications in fault diagnosis. For example, the literature includes example of applications in the context of rolling element bearings (Mechefske and Mathew, 1992; Moosavian et al., 2013; Tian et al., 2016) and gears (Lei and Zuo, 2009; Gharavian et al., 2013).

Enhanced versions of the basic KNN algorithms have been gradually introduced to boost its classification performances and to overcome some of its limitations, such as the computational load it requires to process large-sized datasets. For instance, Appana et al. (2017) introduce a new type of metric which augments the information provided by the distance between sample pairs with their relative densities. Also, Lei et al. (2009) apply a combination of weighted KNN (WKNN) classifiers to fault diagnosis of rolling bearings in order to cope with the problem of data instances belonging to different classes overlapping in the feature space. Finally, in Dong et al. (2017) and (Wang and Ma, 2014), KNN was optimized with the particle swarm algorithm (Kennedy and Eberhart, 1997) to alleviate the storage requirements of the former.

Overall, KNN and its enhanced versions can be considered as relatively effective algorithms for fault diagnosis, especially because of their simplicity and interpretability. Their main limitations stand in the high computational cost and their considerable sensitivity to noise.

##### 3.1.2.2 Prognosis

Generally, prognosis is a more challenging problem than diagnosis and therefore effective methods in this context are less simple to find. Below, we list some of the most interesting applications of ANNs, SVMs, and DTs to fault prognosis. KNNs are not as widespread as in fault diagnosis and their application is not common in RUL estimation.

###### 3.1.2.2.1 Artificial Neural Networks

Two of the first attempts of applying ANNs to fault prognosis problems are introduced in Shao and Nezu (2000) and Gebraeel et al. (2004). Both approaches are proposed in the context of bearings RUL prediction. In Shao and Nezu (2000), a three-layer neural network is used to forecast the value of the bearing health indicator. In Gebraeel et al. (2004) several fully-connected models are trained on either individual or on clusters of similar bearing features. Both methods use manually extracted statistical features as input of the corresponding ANNs. More recent approaches include, for example, Elforjani and Shanbr (2018) and Teng et al. (2016). The first work proposes a comparative study of the performance of SVM, Gaussian Processes (Rasmussen, 2003) and ANNs for RUL estimation from features extracted from acoustic emission signals. The study reveals that the proposed ANN is the best performing model for the RUL prediction task under consideration. In Teng et al. (2016), ANNs are used to provide short-term tendency prediction of a wind turbine gearbox degradation process. The approach is validated by a series of experiments on bearing degradation trajectories datasets, showing good RUL prediction performances.

###### 3.1.2.2.2 Support Vector Machines

SVM-based methods have been extensively applied to fault prognosis tasks. Huang et al. (2015) provide an extensive review of the most relevant techniques employing SVM-related approaches in the context of RUL prediction. Application examples include RUL estimation of bearings (Sun et al., 2011; Chen et al., 2013; Sui et al., 2019), lithium-ion batteries (Khelif et al., 2017; Wei et al., 2018; Zhao H. et al., 2018; Zhao Q. et al., 2018) and aircraft engines (Ordóñez et al., 2019). For instance, in Wei et al. (2018) Support Vector Regression (SVR) is used to provide a state-of-health state-space model capable of simulating the battery aging mechanism. Comparison of the performances provided by an ANN-based model of the same type shows the superiority of the proposed approach over its neural network-based counterpart. In the context of bearings fault prognosis, Sun et al. (2011) introduce a multivariate SVM for life prognostics of multiple features that are known to be tightly correlated with the bearings’ RUL. The proposed method shows good prediction performance and leverages the ability of SVM of dealing with high-dimensional small-sized datasets.

###### 3.1.2.2.3 Decision Trees

DTs and RFs have also been applied to fault prognosis, in particular in the contexts of RUL estimation of bearings (Satishkumar and Sugumaran, 2015; Patil et al., 2018; Tayade et al., 2019), lithium-ion batteries (Zheng H. et al., 2019; Zheng Z. et al., 2019) and turbofan engines (Mathew et al., 2017). In Patil et al. (2018), the authors train a RF to perform RUL regression by using time-domain features extracted from the bearings vibration signals. The model is evaluated on the dataset provided by IEEE PHM Challenge 2012 (Ali et al., 2015), showing improved results than previous benchmarks. One further example is provided by Satishkumar and Sugumaran (2015), who cast the RUL estimation problem into a classification framework. In particular, statistical features in the time domain are extracted from five different temporal intervals from normal condition to bearing damage. A DT is then used to classify new data into one of these intervals, resulting in about 96% accuracy.

#### 3.1.3 Discussion

##### 3.1.3.1 Dependency on Feature Extraction

Traditional ML algorithms have been widely applied both to fault diagnosis and fault prognosis tasks. They present the relevant advantage of combining rather good performances and a relatively high degree of interpretability. On the other hand, most of them rely on good quality features that have to be carefully extracted and selected by human experts. This dependency on the feature extraction step limits the potential of traditional ML methods and imposes a strong inductive bias in the learning process. As we discuss in the next section, “deep” algorithms can extract information directly from raw data and can often improve the generalization performances of traditional ML approaches.

##### 3.1.3.2 Model Selection

It is important to observe that it is not possible to identify a specific algorithm, among those discussed above, that clearly outperforms the others in all possible settings. Selecting a specific technique highly depends on the requirements and characteristics of the PHM problem at hand. For example, a black-box ANN approach might be more suitable when one is mainly interested in performances and less in interpretability, SVMs can be useful in the low-data regime and DTs can be a sensible choice if interpretability is prioritized. Ultimately, the final algorithm is often chosen by calculating a set of performance metrics for each candidate technique and selecting the method providing the highest scores. Some standard example of these measures are accuracy, precision, Recall, F1 Score, Cohen Kappa (CK), and Area Under Curve (AUC). A description of these metrics can be found, for instance, in Bashar et al. (2020).

##### 3.1.3.3 Overfitting

The long-standing problem of overfitting (or over-training) is a well-known pathology affecting data-driven approaches. In essence, it stems from the imbalance between model capacity and data availability. If on one hand, the adoption of ML techniques can be significantly beneficial in PHM, on the other hand, it also requires to think about effective solutions to contrast overfitting in order to fully exploit the advantages of data-driven approaches. In the context of PHM applications, a key requirement for the deployment of a given ML algorithm stands indeed in the robustness of its performances when data different from the training ones kick in. Although algorithm-specific techniques exist to tackle overfitting, held-out-cross validation (Hastie et al., 2001) is probably the most popular one and can be used independently on the particular ML algorithm (see, for instance, Gebraeel et al., 2004), for ANNs (Islam et al., 2017), for SVMs (Abdallah et al., 2018), for decision trees and (Tian et al., 2016) for KNN).

As regards DTs, overfitting is typically tackled by pruning the tree in order to prevent it to merely memorize the training set and improve performances on unseen data (Praveenkumar et al., 2018). Random forests have also been used for the same purpose (Yang et al., 2008). They consist of ensembles of DTs and one of their main benefits is to mitigate the overfitting tendency of standard DTs.

A widely used strategy to contrast over-training in SVMs is to introduce a set of so-called slack variables in order to allow some data instances to lie on the wrong side of the margin (Hastie et al., 2001). The extent to which this class overlapping effect is permitted is regulated by a regularization constant *C*. Furthermore, the smoothness of the margin can be adjusted by appropriately tuning the hyperparameters of the kernel. Sun et al. (2016a), for instance, use cross validation to find optimal values of the constant *C* and of the gaussian kernel width parameter.

In ANNs, the effects of overfitting get increasingly more pronounced as the number of hidden layers increases (Samanta, 2004). Two typical strategies to alleviate its impact are early stopping and regularization. The first consists in stopping the training phase once the first signs of over-training kick in. The second introduces a penalizing term in the loss function (typically in the form of $L_{2}$ or $L_{1}$ norms on the network weights) to keep the values of the weights as small as possible. In Ayhan et al. (2006) for instance, the authors use early-stopping by arresting the training phase once the validation error keeps increasing for a specific number of epochs.

Finally, the KNN algorithm yields different performances depending on the value of *k*. Small values of *k* result in very sharp boundaries and might lead to overfitting. On the other hand, large *k*s are more robust to noise but might result in poor classification performances. This hyperparameter is then typically chosen via cross-validation by selecting the best performing value among a set of candidates. In Gharavian et al. (2013), for instance, *K* is varied from 1 to the number of the training samples.

### 3.2 The Deep Learning Revolution

Most of the methods we have discussed so far are characterized by relatively “shallow” architectures. This aspect results in two main consequences: first, their representational power can be fairly limited and second, their input often consists of high-level features manually extracted from raw data by human experts.

DL is a quite recent class of ML methods that provide a new set of tools that are able to cope with the aforementioned shortcomings of traditional approaches. Essentially, DL techniques arise as an extension of classical ANNs. DL models, in their simplest form, can be seen as standard ANNs with the addition of multiple hidden layers between the network’s input and output. An increasingly large corpus of empirical results has shown that these models are characterized by a superior representational power compared to shallow architectures. Once deep networks are trained, their inputs pass through a nested series of consecutive computations, resulting in the extraction of a set of complex features that are highly informative for the task on interest. This characteristic is one of the hallmarks of DL and can be seen as one of the key factors of its success.

In light of its improved representational power, its ability to automatically extract complex features, its dramatic achievements across different engineering fields and its multiple dedicated freely available software libraries (Jia et al., 2014; Abadi et al., 2016; Theano Development Team, 2016; Paszke et al., 2019), DL has the potential to provide effective solutions also in the context of PHM applications. Big data handling, automated end-to-end feature extraction from different data structures (e.g., images, time-series) and improved generalization are some of the targets on which DL models can make a difference compared to traditional ML approaches.

In the following, we introduce some of the most popular DL techniques used in PHM. Specifically, we focus on Autoencoder (AE) architectures, Convolutional Neural Networks (CNNs), Recurrent Neural Networks (RNNs) and some of their variants and combinations. For each model, we list some interesting applications both in the context of fault diagnosis and prognosis.

#### 3.2.1 Methods and Techniques

##### 3.2.1.1 Autoencoders

AEs, in their simplest form, consist of feed-forward neural networks that are trained to output a reconstructed version of their input. They are composed of two sub-networks, namely an encoder and a decoder. The encoder, h, implements a mapping from the input space to a typically lower-dimensional space. More concretely, we have:

$$h=\psi \left(W_{1}x+b_{1}\right)$$ (3)

where $x\in {\mathbb{R}}^{d}$ is the input vector, *ψ* is the activation function and $W_{1}\in {\mathbb{R}}^{q\times d}$ and $b_{1}\in {\mathbb{R}}^{q}$ are the parameters of the encoder. The decoder implements a mapping from the embedding to the input space in order to reconstruct the original input vector. In formulas:

$$\tilde{x}=\psi \left(W_{2}h+b_{2}\right)$$ (4)

where $\tilde{x}\in {\mathbb{R}}^{d}$ is the reconstructed input vector and $W_{2}\in {\mathbb{R}}^{d\times q}$ and $b_{2}\in {\mathbb{R}}^{d}$ are the parameters of the decoder. Given a dataset of *N* data instances ${\left\{x_{i}\right\}}_{i=1}^{N}$, the accuracy of the model can be measured with, for example, the Root-Mean-Squared-Error (RMSE), which evaluates the reconstruction error made by the autoencoder:

$$\mathrm{RMSE}\left(\theta \right)=\sqrt{\frac{1}{N}\overset{N}{\underset{i=1}{\sum }}{\left(x_{i}-{\tilde{x}}_{i}\left(\theta \right)\right)}^{2}}$$ (5)

**FIGURE 5:**
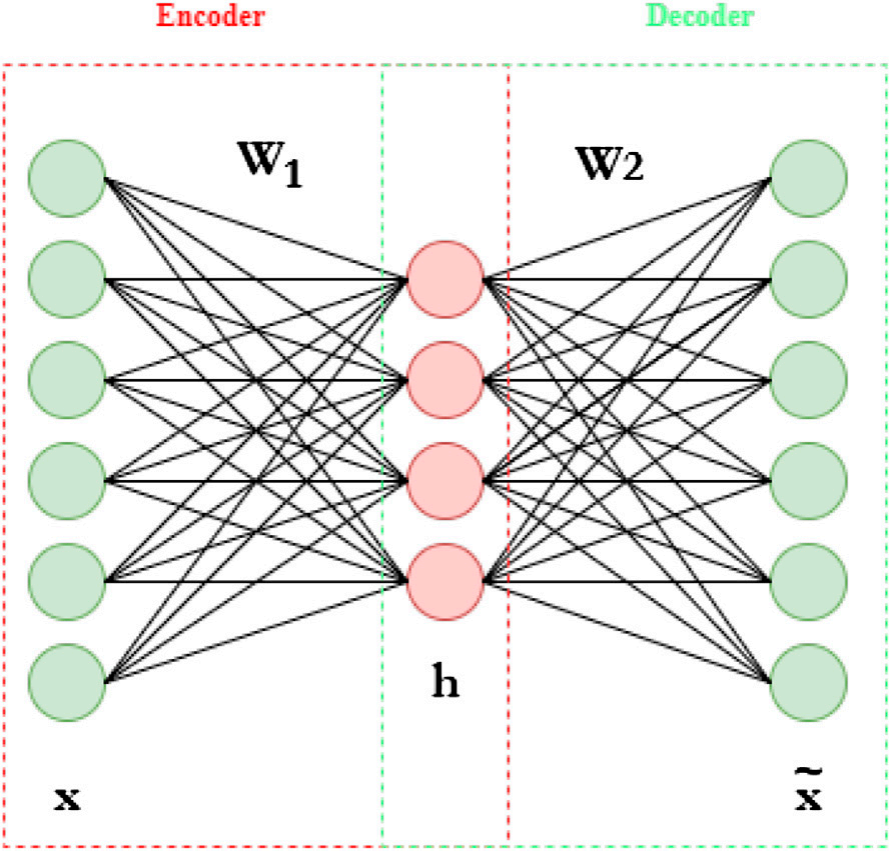
Typical Autoencoder architecture.

In the equation above, the symbol *θ* has been used to indicate the parameters of the network, i.e., $W_{1},\;W_{2},\;b_{1},\;b_{2}$. The value of the parameters is found by minimizing the RMSE w. r.t the parameter *θ* of the model. Figure 5 shows an illustration of the typical AE architecture.

Note that the model assumes a so-called bottle-neck shape, characterized by an embedding space with a lower dimension than the input space. By setting q<d, we can force the algorithm to find a more expressive representation of the input by getting rid of redundant pieces of information and keep only the most relevant ones for the reconstruction purpose. It is important to point out that here we have limited our description to a one-hidden-layer architecture for the sake of simplicity. However, deep models can be simply obtained by consecutively stacking multiple hidden layers. following the bottle-neck architecture.

There exists several more powerful extensions of the basic AE discussed before. Some examples include Sparse AEs (SAEs) (Ng et al., 2011), denoizing AEs (DAEs) (Vincent et al., 2008) and variational AEs (VAEs) (Kingma and Welling, 2013). Sparse AEs regularize the standard AE loss function with an additional term that forces the model to learn sparse features. This regularization term can be, for instance, the $L^{1}$ norm of the activations:

$$\mathrm{Loss}\left(\theta \right)=\mathrm{RMSE}\left(\theta \right)+\lambda \underset{i}{\sum }|h_{i}|,$$ (6)

where $h_{i}$ is the *i*th component of the embedding h. Alternatively, one can consider the KL divergence between the average *i*th activation and a small sparsity parameter α, yielding the following loss:

$$\mathrm{Loss}\left(\theta \right)=\mathrm{RMSE}\left(\theta \right)+\lambda \underset{i}{\sum }KL\left(\alpha ||\rho_{i}\right),$$ (7)

where $\rho_{i}={\sum^{\text{​}}}_{j}^{m}h_{i}\left(x_{j}\right)$ and *m* is the number of training examples.

DAEs take as input corrupted version of the data and aim to output a reconstructed version of the original uncorrupted data. The assumption is that the algorithm is forced to select only the most informative part of the input distribution in order to recover the uncorrupted data instance.

VAEs differ from the previous AE techniques since they belong to the class of generative models. They aim at learning a parametric latent variable model through the maximization of a lower bound of the marginal log-likelihood of the training data. The goal of these approaches is to provide a way to learn a so-called disentangled representation of the latent space, i.e., a representation where the most relevant independent factors of variations in the data are decoupled amd clearly separated. To conclude this part it is worth mentioning that it is possible to design autoencoders where the encoder and the decoder are not limited to simple feed-forward neural networks but can also assume the form of CNNs and RNNs. We discuss these methods later within the section.

##### 3.2.1.2 Convolutional Neural Networks

CNNs are some of the most successful and widely applied DL models. They reached the peak of their popularity thanks to their state-of-the-art performances in CV tasks, such as IR, pose estimation and object tracking. They have also been successfully applied in the contexts of NLP, Reinforcement Learning and time-series modeling. Their design draws inspiration from the organization of animal visual cortex (Hubel and Wiesel, 1968). Indeed, it turns out that single cortical neurons fire in response of stimuli received from relatively narrow regions of the visual field called receptive fields. Furthermore, neurons that are close to each other are often associated with similar and partially overlapping receptive fields, allowing them to map the whole visual field. These properties are useful to recognize specific features in natural images independently of their location.

CNNs implement these concepts by modifying the way computations are usually performed in standard feed-forward neural networks. In particular, CNNs convolve the input image with filters composed of learnable parameters. These parameters are trained to automatically extract features from the image in order to perform the task specified by a final loss function.

**FIGURE 6:**
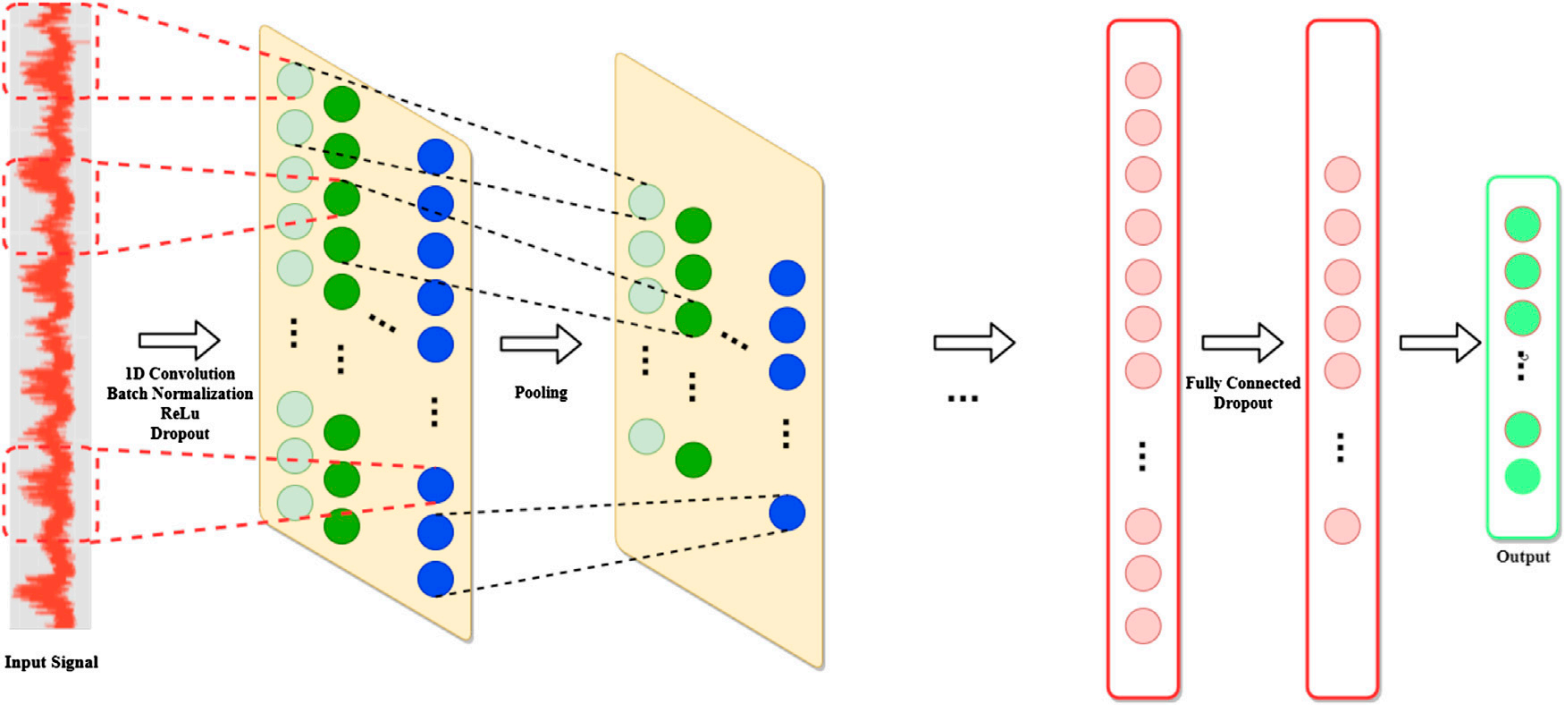
Typical 1D-CNN architecture. Adapted from Jiao et al. (2020).

The standard CNN model shown in Figure 6 is composed of a set of elementary consecutive blocks. First, the input layer defines the data structure. A convolutional layer follows the input layer and performs the convolution operation over the input data. The size of the filters depend on the input structure. Two-dimensional filters are used for grid-like inputs, whereas, one-dimensional filters are used for time-series. Each filter has a user-specified size, which defines its receptive field. Batch normalization (Ioffe and Szegedy, 2015) is often applied right after the convolutional module in order to reduce the so-called covariate shift phenomenon and introduce a regularization effect. Then, a point-wize nonlinear activation function (e.g., ReLU) is applied.

The convolutional layer is then followed by a so-called pooling layer, whose role is to reduce the number of parameters by sub-sampling the filtered signals. One common strategy to perform this operation is called max-pooling and consists of extracting only the maximum value of a fixed-sized batch of consecutive inputs.

Several instances of convolutional and pooling layers are typically alternated through the network. The final filtered signals are then flattened and fed into a sequence of fully-connected layers that map them into the output layer. The dropout (Srivastava et al., 2014) technique can be used both between the fully connected and the convolutional layers in order to contrast overfitting.

##### 3.2.1.3 Recurrent Neural Networks

RNNs form another class of DL methods that has achieved impressive results in a wide variety of ML fields. In particular, RNNs are particularly effective in processing data characterized by a sequential structure. These types of data are widespread in fields such as NLP, Speech Recognition, Machine Translation, Sentiment Analysis to name a few, where recurrent architectures have been employed successfully. Given their particular suitability in analyzing sequential data, it is not surprising that RNN models have been widely applied in the context of PHM applications. We review some of these applications later in this section.

**FIGURE 7:**
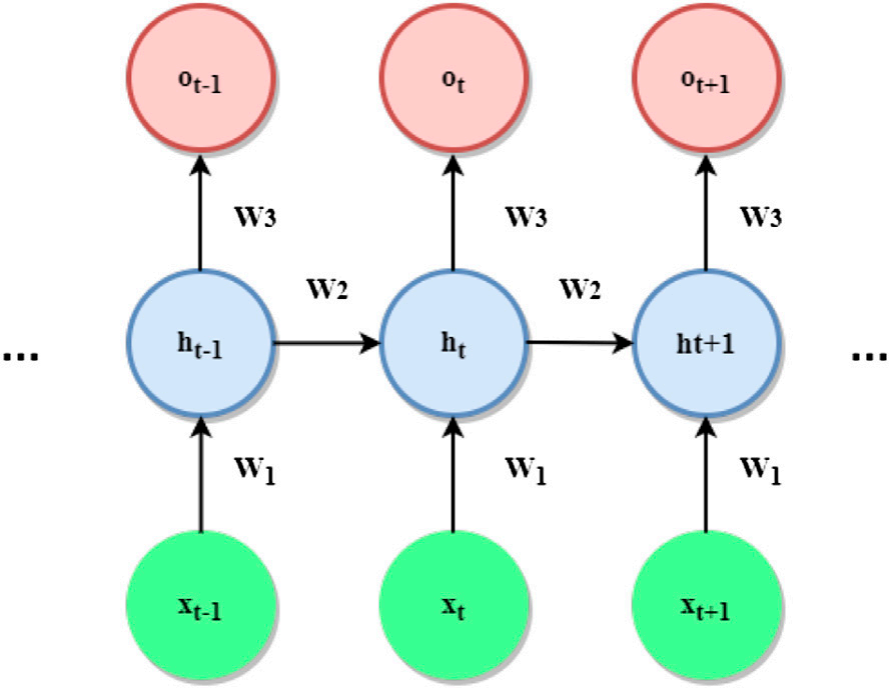
Most elementary RNN architecture.

The architecture of the simplest possible recurrent model is shown in Figure 7.

Given a sequential input vector $x=\left[x_{1},\ldots ,x_{t},\ldots ,x_{T}\right]$, where $x_{t}\in {\mathbb{R}}^{d}$ at each time-step the RNN shown above performs the following operations:

$$\begin{array}{c} h_{t}=\psi_{1}\left(W_{1}x_{t}+W_{2}h_{t-1}+b_{1}\right)\\ o_{t}=\psi_{2}\left(W_{3}h_{t}+b_{2}\right) \end{array}$$ (8)

where, $W_{1}$, $W_{2}$, $W_{3}$, $b_{1}$, $b_{2}$ are the parameters of the model, $\psi_{1}$ and $\psi_{2}$ are activation functions, $h_{t}$ is the so-called hidden state at time *t* and $o_{t}$ is the output at time *t*. Predictions are performed at each time step by mapping the current hidden state to the output. $o_{t}$, through a nonlinear activation. The hidden state is constantly updated at each iteration by combining the previous hidden state and the current input. This allows us to store past information and propagate it over time through the network. The basic architecture described above, however, suffers from the so-called vanishing gradient problem. This phenomenon is caused by the structure of simple RNNs which typically perform the composition of the same function sequentially at each time step. As shown by Bengio et al. (1994), this results in increasingly small magnitudes associated with the gradients of long term interactions. To cope with this problem, a number of refinements have been introduced to the elementary architecture discussed before. The most popular ones are arguably the Long-Short-Term-Memory (LSTM) (Hochreiter and Schmidhuber, 1997), Bidirectional RNNs (Bi-RNN) (Schuster and Paliwal, 1997) and Gated-Recurrent Units (GRUs) (Cho et al., 2014). These techniques have been largely applied, over the last few years, to PHM, both for diagnosis and prognosis tasks. Current state-of-the-art methods in NLP complement the aforementioned recurrent architectures with the so-called attention mechanism (Devlin et al., 2018), which has resulted in significant performance improvements. Despite its success in NLP and related fields, attention-based networks do not find many applications in PHM, indicating a probably fruitful research direction.

#### 3.2.2 Diagnosis

##### 3.2.2.1 Autoencoder

AEs provide a first example of how DL methods can overcome some of the limitations of classical approaches. Indeed, typically AEs are used to automatically extract complex and meaningful features from raw data or to obtain more informative representations of a set of already extracted features. AEs have been applied to data gathered from several machines and industrial components, such as rolling element bearings (Jia et al., 2016; Liu et al., 2016; Lu et al., 2016; Jia et al., 2018), gearboxes (Jia et al., 2018), electrical generators (Michau et al., 2017; Michau et al., 2019), wind turbines (Yang et al., 2016), chemical industrial plants (Lv et al., 2017), induction motors (Sun et al., 2016b), air compressors (Thirukovalluru et al., 2016), hydraulic pumps (Zhu et al., 2015), transformers (Wang et al., 2016), spacecrafts (Li and Wang, 2015) and gas turbine combustors (Yan and Yu, 2019).

As mentioned before, AEs are often used in combination with other classifiers, such as simple softmax classifiers (Liu et al., 2016), feed-forward neural networks (Sun et al., 2016b), RFs (Thirukovalluru et al., 2016) and SVMs (Sun et al., 2016b; Lv et al., 2017). In Sun et al. (2016b), feed-forward NNs trained on top of the features learned by the AE model provide excellent classification results in terms of fault diagnosis accuracy. An SVM trained on the same features performs only slightly worse. Liu et al. (2016) propose a combination of stacked SAEs and a softmax classifier for element bearings fault diagnosis. Short-time-Fourier transformed raw inputs undergo several nonlinear transformations implemented by the sparse AEs. The resulting features are fed into a softmax classifier which outputs the classification results.

Lu et al. (2016) compare the features extracted by stacked DAEs with some manually extracted features. The comparison is based on the fault classification accuracies provided by an SVM and a RF model trained on top of the two classes of features. The results show that the first set of features possess a larger discriminative power for the task under consideration.

Another interesting application of AEs is shown in the work of Jia et al. (2016). Here, the nonlinear mapping implemented by deep AEs is exploited to pre-train an ANN which is in turn used to perform fault diagnosis both on rolling element bearings and planetary gearboxes. More specifically, the weights between two hidden layers are initialized by training an AE to minimize the reconstruction error of the input values specified by the first hidden layer. With this pre-training strategy, the feature extraction ability of AEs is used to encode relevant properties of the data directly into the ANN weight configuration.

AE architectures can also be used to estimate a health indicator which measures the “distance” of a test data point to the training healthy class (Michau et al., 2017; Michau et al., 2019; Wen and Gao, 2018). For example, in the work of Michau et al. (2019) a system comprising of an AE and a one class-classifier is trained with only healthy data to assess the health state of a complex electricity production plant. In this work, both AE and one-class classifier have the structure of a particular type of neural network called Extreme Learning Machine (ELM). ELM-based AEs have been also successfully employed in Michau et al. (2017) and Yang et al. (2016), among others.

##### 3.2.2.2 Convolutional Neural Networks

CNNs are particularly advantageous in the context of fault diagnosis since they implement the feature extraction and classification tasks in an end-to-end fashion. Moreover, they can be applied to several data structures, including both time-series and images (Jiao et al., 2020). A common strategy to employ 2D-CNNs^6^ in PHM applications is to feed these models with image-like data. This poses the problem of how to convert sensor measurements, which are typically in the form of multivariate time-series, into a grid-like structure. Examples of this procedure can be found, for example, in Ding and He (2017), Sun et al. (2017), Guo et al. (2018b), Wen et al. (2018), Cao et al. (2019), Islam and Kim (2019a), Li et al. (2019a), Wang et al. (2019). Most of these works employ popular signal processing techniques to perform the two-dimensional mapping. In particular, Li et al. (2019a) use the S-transform to map bearing vibrational data into a time-frequency representation. Similarly, in Ding and He (2017), Sun et al. (2017), Guo et al. (2018b), Cao et al. (2019), Islam and Kim (2019a) transformations based on the wavelet transform are used to process data gathered from bearings, rotating machinery and gears. An additional strategy is proposed in Wen et al. (2018), where the following mapping is applied to convert time-series data into two-dimensional images:

$$P\left(j,k\right)=\text{round}\left\{\frac{L\left(\left(j-1\right)\times M+k\right)-\text{Min}\left(L\right)}{\text{Max}\left(L\right)-\text{Min}\left(L\right)}\times 255\right\},$$ (9)

where the input signal is a vector of size $M^{2}$, L(j) is signal magnitude at the *j*th time step and P(j,k) is the intensity of the (j,k) pixels in the output image. This technique has been applied to data extracted from rolling element bearings and hydraulic and centrifugal pumps resulting in nearly optimal fault classification accuracy in all three cases.

Another class of methods applies CNNs directly to image data, thus leveraging the great success of these architectures in CV tasks. For example, Janssens et al. (2018); Jia et al. (2019) use CNNs to perform fault diagnosis of rotating machinery based on infrared thermal videos and images respectively. Yuan et al. (2018) propose a method that fuses features extracted from different data structures, including infrared images, for CNN-based fault classification of a rotor system.

Alternatively to 2D-CNNs, 1D-CNNs can be used to directly process time-series data. The literature contains a large number of examples that propose to apply 1D-CNN to bearing (Eren, 2017; Chen et al., 2018; Eren et al., 2019; Qin et al., 2019; Xueyi et al., 2019) and gears (Jing et al., 2017; Yao et al., 2018; Han et al., 2019b) fault diagnosis. Chen et al. (2018), for instance, propose a novel DL model, based on the popular Inception architecture (Szegedy et al., 2015) and a particular type of dilated convolution (Holschneider et al., 1990). The model is trained with data generated from artificial bearing damages and achieves very good performances on real data. The proposed method is pre-processing-free since it takes as input raw temporal signals directly.

The ability of CNN architectures to extract features in an end-to-end manner is tested in Jing et al. (2017). Here, the authors compare the quality of these features with a number of benchmarks consisting of conventional feature engineering approaches. The results show the superiority of the feature-learning pipeline implemented by CNNs over manual feature extraction.

Finally, CNN have also been applied to generate health indicators and to estimate the degradation trend of rolling bearings (Guo et al., 2018a; Yoo and Baek, 2018). In Yoo and Baek (2018), for instance, the authors apply a continuous wavelet transform to the data and feed the resulting two-dimensional images into a 2D-CNN which, in turn, outputs the health indicator.

##### 3.2.2.3 Recurrent Neural Networks

RNNs have been mainly used for fault prognosis and only a relatively small number of works focus on their application to fault diagnosis. Some examples are (Li et al., 2018a; Li et al., 2018b Qiu et al., 2019) for bearings (Zhao H. et al., 2018; Zhao Q. et al., 2018 Yuan and Tian, 2019), for chemical processes control [see Tenessee Eastman dataset (Chen, 2019)] and (Lei et al., 2019) for wind turbines.

These methods can be divided into two categories: “RNN + classifier” and end-to-end approaches. The works of Li et al. (2018a, 2018b) and Yuan and Tian (2019) belong to the first category. The first employs an LSTM-based architecture to extract informative features from the input data. The so-obtained features are then fed into a softmax classifier that performs fault classification. Yuan and Tian (2019) use a GRU network to obtain dynamic features from several sub-sequences extracted from the raw signals. Multi-class classification is performed by a final softmax layer fed with the features obtained by the GRU module.

Zhao H. et al., 2018; Zhao Q. et al., 2018 Qiu et al. (2019); Lei et al. (2019) use RNN architecture in an end-to-end manner. For instance, Qiu et al. (2019) use a variant of Bi-LSTMs specifically designed to process long-term dependencies, to directly classify fault types. The network is trained with a set of features extracted by means of wavelet packet transform and employs softsign activation functions to contrast the vanishing gradient problem. Another end-to-end approach is proposed in Lei et al. (2019) where the authors use an LSTM-based model for fault diagnosis of a wind turbine. In this work, features are directly extracted by the network and there is no need for manual feature extraction. The proposed method is shown to outperform existing fault diagnosis techniques, such as ANNs, SVMs and CNNs.

##### 3.2.2.4 Hybrid

With hybrid approaches we mean all those methods that combine the benefits provided by AEs, CNNs and RNNs models into single powerful systems.

For example, Li et al. (2019d); Park et al. (2019) propose techniques leveraging the efficacy of AEs in extracting valuable features and the advantages provided by RNN-architectures in analyzing time-dependent data. In Li et al. (2019d), first stacked AEs generate a latent representation of the raw input rotary machinery data. An LSTM network is then used to predict the value corresponding to the 10-th time step in the feature sequence given the previous 9. The reconstruction error between prediction and ground truth value is used to determine if the datum is anomalous or not.

An alternative approach consists in using recurrent models in the form of AEs to better deal with time-series data. In Liu et al. (2018), for instance, a GRU-based DAE is proposed for rolling bearing fault diagnosis. Specifically, the proposed GRU model is used to predict the next period given the previous one. As many such models as the number of faults are trained and classification is performed by selecting the model providing the lowest reconstruction error.

CNN-based architectures can also be combined with other types of networks for the purpose of fault diagnosis. In Liu et al. (2019b), for instance, a one-dimensional convolutional-DAE is proposed to extract features from bearing and gearbox data. This model is given corrupted time-series as input and its goal is to clean and reconstruct them at the output level. The so-learned features are then fed into an additional CNN model that performs the classification task.

In Zhao et al. (2017), Pan et al. (2018), Xueyi et al. (2019), the combination of CNNs and RNNs is investigated. For example, in Xueyi et al. (2019) a 1D-CNN and a GRU network are used to extract discriminative features from acoustic and vibration signals respectively. The so-obtained features are then concatenated and fed into a softmax classifier which performs gear pitting fault diagnosis. This hybrid method is shown to outperform CNN and GRU applied individually to the same data.

Pan et al. (2018), instead, proposes a method fusing a 1D-CNN and an LSTM network into a single structure. The LSTM takes as input the output of the CNN and performs fault diagnosis over bearing data. The proposed algorithm provides nearly optimal performances on the test set.

#### 3.2.3 Prognosis

##### 3.2.3.1 Autoencoder

AEs are typically used in combination with other regression techniques for the purpose of fault prognosis. The literature contains examples of AE-based techniques applied to RUL estimation of bearings (Ren et al., 2018; Xia et al., 2019), machining centers (Yan et al., 2018), aircraft engines (Ma et al., 2018) and lithium-ion batteries (Ren et al., 2018b). The role of AEs in all the above references is to perform automatic feature extraction to facilitate the work of regression or classification methods used for health state assessment or RUL estimation. Xia et al. (2019), for example, utilize a DAE and a softmax classifier trained on top of the AE embedding to classify the inputs into different degradation stages. Then, ANN-based regressors are used to model each stage separately. The final RUL is obtained by applying a smoothing operation to all the previously computed regression models.

In Ma et al. (2018), AEs are used in a similar manner. The authors propose a system composed of a DAE, a SAE and a logistic regressor to predict the RUL on an aircraft engine. The first AE module generates low-level features which are in turn fed into the second AE model which outputs a new set of high-level features. Finally, the logistic regressor predicts the RUL based on the features extracted by the second AE.

##### 3.2.3.2 Convolutional Neural Networks

CNN architectures have been extensively explored also for fault prognosis. These methods have been mainly applied to open-source evaluation platforms such as the popular NASA’s C-MAPSS dataset (Saxena and Goebel, 2008) for aero-engine unit prognostics (Babu et al., 2016; Li et al., 2018a; Li et al., 2018b Wen et al., 2019a) and the PRONOSTIA dataset (Ali et al., 2015) for bearings health assessment (Ren et al., 2018a; Zhu et al., 2018; Li et al., 2019c; Wang et al., 2019b; Yang et al., 2019).

In Li et al., 2018a; Li et al., 2018b a 1D-CNN model is used to predict the RUL on the C-MAPSS dataset. Data are first chunked in fixed-length windows and then directly fed into the network without any pre-processing step. Despite the relative simplicity of the employed architecture, the proposed technique is able to provide pretty good prediction results, especially in proximity of the final failure.

In Wen et al. (2019a) the authors build upon the work of Li et al., 2018a; Li et al., 2018b and propose a novel CNN model for RUL estimation which draws inspiration from the popular ResNet architecture (He et al., 2016). The proposed technique is shown to outperform traditional methods such as SVMs, ANNs, LSTM and the model proposed by Li et al., 2018a; Li et al., 2018b in terms on RUL mean and standard deviation on the C-MAPSS dataset.

In the context of bearing fault prognosis, Ren et al. (2018a) propose a new approach based on manual feature extraction and CNNs for RUL estimation. First, a new method for feature extraction is proposed to generate a feature map which is highly correlated with the decay of bearing vibration over time. This feature map is then fed into a deep 2D-CNN which outputs the RUL estimate. Linear regression is then used as a smoothing method to reduce the discontinuity problem in the final prediction result. Experiments show that the proposed method is able to provide improved prediction accuracy in bearing RUL estimation.

##### 3.2.3.3 Recurrent Neural Networks

The application of RNN architectures to fault prognosis have been explored on various industrial components such that lithium-ion-batteries (Zhang et al., 2018), gears (Xiang et al., 2020), fuel cells (Liu et al., 2019a), and on the C-MAPSS dataset (Yuan et al., 2016; Zheng et al., 2017; Wu et al., 2018a; Wu et al., 2018b; Chen et al., 2019; Elsheikh et al., 2019; Wu et al., 2020). One of the most popular RNN-based approaches proposed in the literature is the work of Wu et al. (2018b). The authors first extract dynamic features containing inter-frame information and then use these features to train a vanilla-LSTM model to predict the RUL. An SVM model is employed to detect the degradation starting point. The proposed technique is shown to consistently outperform a standard RNN and a GRU model trained on the same dataset. The remarkable performances of LSTM networks on the RUL estimation task are further confirmed by the work of Zheng et al. (2017). The authors combine LSTM layers with a feed-forward neural network, showing that the proposed approach provides better performances than ANNs, SVM and CNNs. In Xiang et al. (2020), the attention mechanism is used to enhance the performances of an LSTM network on the prediction of the RUL on gears. The aforementioned model, named LSTMP-A, is trained with time-domain and frequency-domain features and its comparison with other recurrent models shows that it provides the best prediction accuracy.

##### 3.2.3.4 Hybrid

Hybrid approaches have been also applied in the context of fault prognosis. For instance, the literature contains examples of AE + RNN (Lal Senanayaka et al., 2018; Deng et al., 2019) and CNN + RNN (Zhao et al., 2017; Mao et al., 2018; Li et al., 2019b) combinations. In Zhao et al. (2017) sensory data from milling machine cutters are processed by a novel technique combining a CNN component and an LSTM network. The CNN is used to extract local features, whereas a bi-LSTM captures long-term dependencies and take into account both past and future contexts. A sequence of fully connected layers and a linear regression layer takes as input the output of the LSTM and predicts the tool-wear level.

Similarly, Mao et al. (2018) combine LSTM and CNN models for feature extraction and RUL prediction. In particular, time-series from the C-MAPPS dataset are first sliced by applying a time-window. The resulting data are then independently fed into an LSTM network and a CNN. The features extracted by these two networks are then combined and further processed by an additional LSTM network and a fully connected layer which predicts the RUL.

Deng et al. (2019) propose a method based on the combination of stacked SAEs and a GRU model. The AE is used for automatic feature extraction and the GRU is used to model the mapping from the features extracted by the AE to the RUL values. The proposed method is applied to the C-MAPPS dataset, showing satisfactory results.

#### 3.2.4 Discussion

##### 3.2.4.1 Dependency on Feature Extraction

One of the key advantages of DL algorithms over traditional ML approaches stands in their lower degree of dependence on the feature extraction step. Their input can consist of either raw data or a set of manually extracted features, depending on the amount of prior information available to the user about the task under consideration.

##### 3.2.4.2 Model Selection

As already discussed for traditional ML algorithms, a universal approach valid for all possible application scenarios does not exist. In general, the nature of the problem dictates which method to utilize. For instance, when the PHM problem at hand involves image data, the usage of 2D CNN might be preferred. On the other hand, when sensor measurements consisting of time-series data have to be analyzed, 1D CNN and RNN architectures are more sensible choices. Ultimately, the final model can be selected by evaluating each candidate on the same metrics mentioned at the end of paragraph 3.1.3.2 and comparing the corresponding scores.

##### 3.2.4.3 Overfitting

As already mentioned before, a larger number of hidden layers is often associated with a higher risk of overfitting. Beyond the techniques already discussed for ANNs (e.g., cross-validation, early-stopping and regularization), deep models can be equipped with more advanced tools to contrast over-training. A popular example is the Dropout technique (Srivastava et al., 2014) which randomly drops neurons from the neural network at training time. Intuitively, this prevents the network to specialize on a particular set of data. Dropout is used, for instance, in Han et al. (2019b) and Wang et al. (2019) with the corresponding parameter fixed at 0.5. Finally, data augmentation can be also used to generate new images by applying simple transformations (e.g., rotation, mirroring, cropping, padding) to the training data. For instance, this technique is applied in Wang et al. (2019) to time-frequency images obtained from bearing accelerometers, in order to increase the size and the level of diversity of the training set.

## 4 Critique and Future Directions

In the previous section, we have discussed some of the most popular DL techniques that have been applied to PHM problems over the last few years. We have compared traditional ML approaches with DL techniques, trying to highlight the strengths of both methods and emphasizing the change of paradigm introduced by the so-called DL revolution.

The goal of this section is to shed some light over a number of open challenges that need to be addressed to bridge the gap between research and industrial applications. We start by briefly discussing some of these open questions and some limitations of DL models that hinder their solution. Then, we discuss some first attempts to cope with these challenges along with some proposals of future investigations. Our goal is to provide the reader with a set of possible fruitful research directions that we consider as valuable candidates to further increase the impact of DL to PHM.

### 4.1 Open Challenges

#### 4.1.1 Reliability and Interpretability

One of the most common criticisms to DL models arises from their black-box nature, i.e., the sometimes opaque mechanism by which they make their decisions. This characteristic of deep models derives from one of the properties that allows them to successfully tackle several different tasks: the complex sequence of nonlinear operations they implement across their deep architectures. A complete mathematical characterization of the behavior of DL models in light of their inherent complexity is very hard to obtain. This negative property of deep networks represents a significant limitation to their deployment in areas such as healthcare, finance, and PM. In these delicate contexts, humans need to have control over their tools and it is not always possible to sacrifice trust and transparency for better performances. It is therefore urgent to enhance the level of interpretability of these models in order to make them fully deployable while minimizing the risks.

However, it is not straightforward to provide a unique definition of the concept of interpretability (Lipton, 2018). DL models can be, for instance, enhanced with complementary functionalities responsible for providing a post-hoc explanation of their actions. Alternatively, one can build some notion of interpretability directly into the models in order to constrain their learning process to align with some inductive biases that we might deem trustworthy. The strategy of providing post-hoc explanations of the model behavior have been widely investigated in CV (Ribeiro et al., 2016; Zhou et al., 2016; Lundberg and Lee, 2017). Few attempts, however, have been made to extend these approaches to time-series data [see for example (Fawaz et al., 2019), (Guillemé et al., 2019)].

Imposing appropriate inductive biases on DL models have been recently identified as a key step to perform unsupervized learning tasks (Locatello et al., 2019a; Locatello et al., 2019b). Some possible inductive biases can derive from a-priori available physical knowledge of the problem under consideration. This complementary information can be incorporated directly into the network architecture or can be used to drive a model toward more meaningful output decisions. We discuss some of these approaches later in this section.

To conclude this discussion, it is worth mentioning that another important requirement for interpretable and transparent models stands in their ability to provide uncertainty estimates about their predictions. Uncertainty can derive both from the intrinsic stochasticity of the task (aleatoric uncertainty) and from the approximations introduced by our imperfect model (parametric uncertainty). Bayesian approaches can in principle deal with uncertainty estimation and their combination with DL methods is a hot research area (Damianou and Lawrence, 2013; Blundell et al., 2015; Garnelo et al., 2018).

#### 4.1.2 Highly Specialized Models

An increasing amount of experimental evidence (Zhang et al., 2017; Beery et al., 2018; Arjovsky et al., 2019) has recently attracted the attention of the scientific community on an additional relevant limitation of deep models: they often tend to learn “shortcuts” instead of the underlying physical mechanisms describing the data. For instance, let’s consider the task of classifying cows and camels based on a training set containing labeled images where cows are mostly found in green pastures and camels in sandy deserts (Beery et al., 2018). Testing our model on images of cows taken in a different environment, such as beaches, leads to a wrong classification decision. Similar generalization deficiencies can be also observed in the context of PHM applications. Typically, labeled data are available only for a single machine; training a model on these data can lead to good performances on a test set extracted from the same machine but to very disappointing results on a similar machine operating at slightly different operating conditions. The variability in the machines’ operational modes can arise from differences in specific choices in their design, or to external factors (e.g., environmental variables such as humidity, temperature, seasonality). Ideally, an efficient model should be able to deal with these factors of variability and provide predictions that are robust to changing operating conditions. On the other hand, the majority of the DL approaches proposed in the literature do not address this point and focus on relatively narrow systems without taking generalization into account. If we really aim at designing “Intelligent” systems that can take decisions following similar cognitive patterns as those characterizing human decision making, we have to provide new solutions to the aforementioned shortcomings.

#### 4.1.3 Data Scarcity

An immediate consequence of using DL models is that, by increasing the depth of the network, the number of parameters associated with it grows accordingly. As a result, finding an optimal weight configuration requires training these networks with very large datasets. In particular, supervised learning approaches are based on the availability of large numbers of labeled data instances for each class under consideration. This aspect poses a significant practical limitation on the application of DL models to the industry domain. In the case of fault diagnosis, for example, it is difficult to find an adequately large number of data for each possible fault. This is mainly because, luckily, faulty data tend to be relatively rare compared to healthy ones. Furthermore, it might also be the case that some faults are not even a-priori known and it is, therefore, impossible to precisely characterize them. This lack of representativeness (Michau et al., 2018) of the training data delineates a very common scenario in practical applications. Two possible alternative approaches can be adopted to cope with it: the first is to design algorithms that are less data-intensive, whereas the second is to generate artificial data that strongly resemble real ones. We discuss some of these methods in the next section.

### 4.2 Possible Solutions

#### 4.2.1 Fusing Deep Learning With Physics

One possible way to cope with the aforementioned challenges is to incorporate information about the physics of the system under consideration into the learning process. DL algorithms, in and of themselves, are not able to capture the primitive causal mechanisms at the basis of the input observations (Pearl, 2019). On the other hand, physical models of complex systems are built from fundamental laws of physics but often rely on relatively strong approximations which result in poor predictive power. Taking prior physics knowledge into account can be helpful in inducing a higher level of interpretability into deep models and in improving their generalization performances. Hybrid models integrating the flexibility of modern data-driven techniques and the transparency of physics models have the potential of overcoming the limitations of the two stand-alone approaches by exploiting their individual strengths.

In the context of PHM, a relatively small number of works have been proposed in this direction. For example, in Chao et al. (2019), a high-fidelity performance model of an aircraft engine is first calibrated on real data by using an Unscented Kalman Filter (Julier and Uhlmann, 1997) and then used to generate unobserved physical quantities that are in turn employed to enhance the input space of a DL model. The results show that the new input space including both observed and virtual measurements contributes in significantly improving the performances of the model.

An alternative way to fuse physics knowledge and data-driven methods is described in Dourado and Viana (2020) and Nascimento and Viana (2019). In these works, well-known physics-based cumulative damage models are complemented by data-driven techniques whose goal is to explain some additional phenomena that the original model is not able to accurately describe. The final model has a sound physical interpretation and provides refinements over the original physics model thanks to its data-driven component.

We conclude this part by noticing that physics knowledge could also be incorporated into deep models directly at the architecture level. Recent research in Graph Neural Networks (Sanchez-Gonzalez et al., 2018; Cranmer et al., (2020)) shows that these kind of models are particularly suitable to encode and exploit prior physics knowledge, for instance, given in the form of Partial Differential Equations over space and time. An example of an industrial application of these models is provided by Park and Park (2019) who use a specific type of GNN to estimate the power generated by a wind farm by modeling the physics interactions between the individual turbines.

#### 4.2.2 Domain Adaptation

The high variability of machines’ operating conditions and the problem of data scarcity motivate the introduction of techniques capable of transferring the knowledge gained from a well-known machine to another for which data are not as abundant. Transfer Learning (TL) is a class of ML methods whose goal is to address this problem. Traditional TL approaches (Yosinski et al., 2014) are based on the following rationale: first, a deep network is trained on a large dataset to perform a specific task. Then, the same network is used to perform a similar task simply by fine-tuning its final layers on a few instances from the new dataset. Recent works in the context of fault diagnosis and fault prognosis have successfully applied this idea on datasets from induction motors (Shao et al., 2019a; Shao et al., 2019b), gearboxes (Cao et al., 2018; He et al., 2019; Shao et al., 2019a; Shao et al., 2019b), bearings (Shao et al., 2019a; Shao et al., 2019b Wen et al., 2019b) and centrifugal pumps (Wen et al., 2019b).

Besides traditional TF methods, unsupervized Domain Adaptation (DA) techniques have also been recently applied to PHM tasks. DA is a sub-field of TF, whose goal is to maximize the performances on the target domain for which only few unlabeled data are available by exploiting a labeled data from the so-called source domain. The two domains are commonly assumed to share similar features even though a model trained on the source domain will usually provide poor performances on the target domain. This is typically due to a distributional shift between the marginal distributions describing the two sets of data. DA techniques have witnessed an increasing attention since the introduction of the so-called adversarial DA methods (Ganin and Lempitsky, 2014; Ganin et al., 2016; Tzeng et al., 2017). These approaches draw inspiration from the training procedure used by the popular Generative Adversarial Networks (GANs) (Goodfellow et al., 2014) to efficiently align source and target domain features in a common latent-space. Several new techniques (Han et al., 2019a; Wang et al., 2019a; Wang and Liu, 2020) based on this class of DA approaches have been recently proposed in the PHM literature. Other references on DA and TF approaches in the context of fault diagnosis can be found in the recent review works of Li et al. (2020) and Zheng H. et al., 2019; Zheng Z. et al., 2019.

#### 4.2.3 Artificial Data Generation

Generative models such as GANs and VAEs have achieved impressive results in generating photo-realistic artificial data in the context of CV. However, the task of generating realistic problem-specific time-series data is still relatively unexplored compared to artificial image generation. Unsurprisingly, existing approaches in this context make large use of GANs. In Donahue et al. (2018), for instance, GANs are used for music and speech synthesis. In Nik Aznan et al., (2019), Haradal et al. (2018), and Hyland et al., (2017) the authors propose new GAN-based methods that generate medical data such as electroencephalographic (EEG) brain signals, and time-dependent health parameters of patients hospitalized in the Intensive Care Unit (ICU). The recent method proposed by Yoon et al. (2019) provides new state-of-the-art performance for realistic time-series generation.

The benefits of such approaches in the context of PHM could be significant. One of their most direct application is to perform data augmentation in order to tackle to problem of lack of representativeness and therefore improving the performance of data-intensive DL models. To the authors’ knowledge, only a small number of works have started exploring this idea and some first interesting results have already been produced (Mao et al., 2019; Shao et al., 2019a; Shao et al., 2019b Wang et al., 2019).

## 5 Discussion

PM, as a key player in the Industry 4.0 paradigm, strongly relies on some of the most recent advances in hardware technology, communication systems and data science. Among them, DL techniques have gained popularity over the last few years in light of their excellent performances in processing complex data in an end-to-end fashion. In this review, we have described several applications of these methods to PHM. In particular, we have discussed the advantages they introduce over traditional ML techniques, stressing on their improved representational power and their ability to automatically extract informative features from data. Despite its great success, DL presents some shortcomings that limit its large-scale deployment in industrial applications. Its low level of interpretability, its generalization deficiencies and its data-intensive nature are some of the main weaknesses DL needs to overcome to close the gap between academia and industrial deployment. In this review, we identified three research areas that we believe could address or alleviate the aforementioned open challenges, namely: physics-enhanced techniques, domain adaptation and artificial data generation. The first aims to improve interpretability by grounding data-driven methods on well-understood physics models of the system under consideration. Furthermore, incorporating prior physics knowledge into DL algorithms can be seen as imposing meaningful inductive biases into the learning process, resulting in improved generalization and reasoning. Domain adaptation provides a set of tools to transfer the knowledge acquired on a well-known industrial component to other similar assets for which data are less abundant. Finally, artificial data generation techniques can be used to cope with the lack of representativeness problem and the data-intensive nature of DL algorithms. Some of these lines of research have already shown interesting results, while others, although very promising, are only in their infancy.

## Author Contributions

LB designed the study and wrote the manuscript. IK contributed to the final version of the manuscript and supervised the project.

## Conflict of Interest

Authors IK and LB are employed by the company CSEM SA.

The authors declare that this study received funding from CSEM SA. The funder was not involved in the study design, collection, analysis, interpretation of data, the writing of this article or the decision to submit it for publication.

## Footnotes

1
https://www2.deloitte.com/content/dam/Deloitte/us/Documents/process-and-operations/us-cons-predictive-maintenance.pdf

2
https://www.isa.org/standards-publications/isa-publications/intech-magazine/2013/feb/automation-it-predictive-maintenance-embraces-analytics/

3
https://www.ge.com/uk/sites/www.ge.com.uk/files/PAC-Predictive-Maintenance-GE-Digital-Full-report-2018.pdf

4
https://www.pwc.be/en/documents/20180926-pdm40-beyond-the-hype-report.pdf

5
https://www.phmsociety.org/sites/phmsociety.org/files/Tutorial_PHM12_Wang.pdf

6 We use the notation “(1D)2D-CNN” to indicate a CNN architecture with (one) two-dimensional filters.
